# Reviving Lonidamine and 6-Diazo-5-oxo-L-norleucine to Be Used in Combination for Metabolic Cancer Therapy

**DOI:** 10.1155/2015/690492

**Published:** 2015-09-06

**Authors:** Diana Cervantes-Madrid, Yair Romero, Alfonso Dueñas-González

**Affiliations:** ^1^Instituto Nacional de Cancerología, 14080 Mexico City, DF, Mexico; ^2^Facultad de Ciencias, Universidad Nacional Autónoma de México, 04510 Mexico City, DF, Mexico; ^3^Instituto de Investigaciones Biomédicas, Universidad Nacional Autónoma de México/Instituto Nacional de Cancerología, 14080 Mexico City, DF, Mexico

## Abstract

Abnormal metabolism is another cancer hallmark. The two most characterized altered metabolic pathways are high rates of glycolysis and glutaminolysis, which are natural targets for cancer therapy. Currently, a number of newer compounds to block glycolysis and glutaminolysis are being developed; nevertheless, lonidamine and 6-diazo-5-oxo-L-norleucine (DON) are two old drugs well characterized as inhibitors of glycolysis and glutaminolysis, respectively, whose clinical development was abandoned years ago when the importance of cancer metabolism was not fully appreciated and clinical trial methodology was less developed. In this review, a PubMed search using the words lonidamine and 6-diazo-5-oxo-L-norleucine (DON) was undertaken to analyse existing information on the preclinical and clinical studies of these drugs for cancer treatment. Data show that they exhibit antitumor effects; besides there is also the suggestion that they are synergistic. We conclude that lonidamine and DON are safe and potentially effective drugs that need to be reevaluated in combination as metabolic therapy of cancer.

## 1. Introduction

Like normal cells, malignant cells have evolved mechanisms to sense external and internal cues in order to maintain cellular homeostasis and survive under different environmental conditions. Both normal and tumor cells efficiently adjust their metabolism in response to the availability of nutrients, energy, and growth factors. The ability to rewire cellular metabolism between anabolic and catabolic processes is crucial for cells to thrive. Thus, cells have developed, through evolution, metabolic networks that are highly plastic and tightly regulated to meet the requirements necessary to maintain cellular homeostasis. The plasticity of these cellular systems is tightly regulated by complex signaling networks that integrate the intracellular and extracellular information. The coordination of signal transduction and metabolic pathways is essential in maintaining a healthy or malignant rapidly responsive cellular state. The importance of the balance between anabolic and catabolic processes is apparent when the metabolic differences between resting and growing cells are studied. Proliferating cells (normal and malignant) rewire their metabolism to promote anabolic processes that synthesize the macromolecules (proteins, carbohydrates, lipids, and nucleic acids) required for generating a daughter cell, whereas in resting cells their metabolism is normally directed towards catabolic processes that provide energy to sustain cellular integrity and function. These processes, therefore, are key to maintaining this balance [1–3].

Until recently, the study of the metabolic alterations in cancer cells was centered in the abnormalities of the glucose metabolism which were recognized more than 90 years ago by Warburg et al. [4]. Early observations on why cancer cells (and highly proliferating normal cells as well) engage onto a less efficient process to generate energy in the form of ATP by no fully oxidizing glucose via its entry into the Krebs cycle were difficult to reconcile with the fact that proliferating cells are in need of high amounts of ATP, especially on the light of evidences suggesting that tumor cells were frequently defective in undergoing oxidative phosphorylation [5]. As the study of normal and tumor metabolism has evolved, there is now evidence that biosynthetic requirements, especially by linking glycolytic activity to macromolecular synthesis, suggest that the major function of enhanced glycolysis in proliferating cells is to maintain constant levels of glycolytic intermediates as macromolecular precursors. This clearly illustrates that increased glycolysis in cancer cells and other proliferating cells provides a selective advantage for growth beyond rapid ATP generation.

On the other hand, glutamine is a nonessential amino acid whose primary functions are to store and traffic nitrogen and carbon between organs. In the body, glutamine accounts for more than 20% of the free amino acid pool in plasma and more than 40% in muscle [6, 7]. Currently, it seems clear that cancer cells do need not only the glucose-derived carbon but also the nitrogen and carbon backbone of glutamine in order to grow and proliferate. In this regard, the Krebs cycle, besides being the major source of energy by providing ATP molecules during full oxidation of substrates, provides biosynthetic precursors in a reaction called cataplerosis. In this process, citrate is used for lipid synthesis whereas oxaloacetate and alpha-ketoglutarate are used to synthesize the nonessential amino acids aspartate, asparagine, glutamate, and proline. To sustain cataplerosis for the Krebs cycle, another process must occur, that is, anaplerosis which can be regarded as the production of oxaloacetate without first passing through Acetyl-CoA. Although pyruvate and some amino acids are known to be anapletoric contributors, glutamine is the major anaplerotic player. The carbons of glutamine are used for the synthesis of the Krebs cycle cataplerotic intermediates, amino acids, and lactate [8–10], and also by being a source of carbons for acetyl-CoA, glutamine is important for the synthesis of fatty acids [11–13]. There is evidence that certain cancer cells use glutamine for nitrogen donation and, in fact, cannot survive if glutamine is not provided but they can, if ammonia is added as a nitrogen source [14]; hence, it seems that nitrogen, not the carbon skeleton, is the most relevant donor function of glutamine for cancer cells. Glutaminolysis therefore is the term derived from the “similarities” of this process with glycolysis [15]. The role of glutaminolysis in cancer cell metabolism was rediscovered by the observation that glutamine withdrawal in contrast to glucose withdrawal was more potent in triggering cell death in Myc transformed cells [16, 17].

In multicellular organisms, cells must be responsive to systemic cues of the physiological state to maintain energetic and cellular stability in addition to sensing the immediate environment. This is achieved through the ability of the cells to sense secreted factors (e.g., cytokines, growth factors, and hormones) that, upon binding to a cell surface receptor, initiate signaling cascades that transduce information and regulate metabolism. Moreover, to ensure the balance between the availability of nutrients and the cellular capacity to use them effectively, cells can also sense intracellular metabolite concentrations to fine-tune the signaling networks independently of the environment. Over the past two decades there are multiple evidences that oncogenic alterations of tumor cells are mechanistically linked or responsible for the altered metabolism of cancer cells [18, 19]. Thus, oncogenes such as* myc*,* K-ras*,* NF-κB*,* HIF-1*,* AKT*,* EGFR*s, and* IGFR*, to mention some, as well as inactivated tumor suppressor genes such as* p53* and* PTEN*, are key players in the process [20–22]. Interestingly these and other oncogenes and tumor suppressor genes directly or indirectly converge onto two highly conserved and crucial pathways, the phosphatidylinositol-3-kinase (PI3K/AKT) and the extracellular signal-regulated kinase-mitogen-activated protein kinase (ERK/MAPK) signaling cascades. The activation of these two signalling pathways rewires malignant cells to acquire an anabolic phenotype to promote anabolism by multiple actions which include direct phosphorylation and regulation of metabolic enzymes, activating and inactivating transcription factors that regulate metabolism as well as modulating a number of regulatory kinases [2]. The PI3K/AKT and ERK/MAPK pathways also exert many of their metabolic actions upon activation of the mTOR complexes, more specifically on the mTOR Complex-1 (mTORC1) which drives ATP-consuming cellular processes necessary for cells to grow and proliferate. mTORC1 regulates not only protein synthesis by inducing mRNA translation and ribosome biogenesis through its canonical substrates S6 kinases (S6Ks) and the inhibitory eIF4E-binding proteins (4EBPs), but it is also known that this complex regulates other major metabolic pathways of the cell, including lipid and nucleic acid synthesis, glycolysis, glutaminolysis, Krebs cycle, and oxidative phosphorylation, further supporting the idea of mTORC1 as a master regulator of metabolism [23–25]. Accordingly, the Ras/Raf/MEK/ERK and PI3K/PTEN/AKT signaling cascades are mutated or aberrantly expressed in most human cancers. Alterations in these pathways also occur by mutations at genes encoding upstream receptors (e.g., EGFR and Flt-3) and chimeric chromosomal translocations (e.g., BCR-ABL), which transmit their signals through these cascades. The fact that these two conserved pathways are commonly altered in most cancers rewire cancer metabolism towards the malignant metabolic phenotype characterized by the anabolic state of tumor cells, aside by inactivating mutations in tumor suppressor genes, whose products that are within or interact with these and other pathways explain why altered metabolism is another hallmark of cancer [26, 27].

## 2. Glycolytic and Glutaminolytic Inhibitors in Development

As the abnormal metabolism of glucose and glutamine is the most studied alterations in cancer, inhibitors of glycolysis and glutaminolysis are in preclinical and clinical development yet none has reached an approved status. Among glycolytic inhibitors there are several classes which target different steps of glycolysis such as (i) glucose transporter (GLUT) inhibitors: phloretin, WZB117, and fasentin; (ii) hexokinase II (HK-II) inhibitors: lonidamine and the glucose analog 2-deoxyglucose (2-DG); (iii) fructose 2,6-bisphosphate (F-2, 6-BP) inhibitor: 3-(3-pyridinyl)-1-(4-pyridinyl)-2-propen-1-one (3PO); (iv) pyruvate analogs: 3-bromopyruvate (3-BrPA); (v) pyruvate kinase M2 (PK-M2) inhibitors: several small-molecule inhibitors in study; (vi) LDH inhibitors: FX11 and oxamate; (vii) monocarboxylate transporters (MCT) inhibitors: *α*-cyano-4-hydroxy-cinnamic acid; and (viii) pyruvate dehydrogenase kinase (PDK) inhibitor: dichloroacetate. Of these, only lonidamine, 2-DG, and lately dichloroacetate have been clinically tested whereas the pyruvate analog 3-bromopyruvate (3-BrPA) has recently entered into clinical phase I testing [65, 66].

Regarding glutaminolytic agents, these are fewer. Among these oldest ones are the (i) glutamine analogs: acivicin, 6-diazo-5-oxo-L-norleucine (DON), azaserine, and azotomycin and (ii) miscellaneous more selective and potent inhibitors: bis-2-(5-phenylacetamido-1,2,4-thiadiazol-2-yl)ethyl sulfide (BPTES) and its analogs. Other agents are ebselen, chelerythrine, apomorphine, and CB-839 [67–70]. Apart from the oldest analogs of glutamine such as acivicin, DON, azaserine, and azotomycin which were clinically evaluated several decades ago and their development abandoned, only CB-839 among the newest analogs has recently reached to phase I clinical trials.

## 3. Lonidamine

### 3.1. Chemistry

Lonidamine, a powerful antispermatogenic agent [71], also known as 1-(2,4-dichlorobenzyl) indazole-3-carboxylic acid, 1-(3,4-dichlorobenzyl)-1H-indazole-3-carboxilic acid, or 1-(2,4-dichlorobenzyl)-1H-indazole-3-carboxylic acid has a molecular weight of 321.1581 and its empirical formula is C_15_H_10_Cl_2_N_2_O_2_. Lonidamine is a powder with an off-white to yellow appearance, soluble in 5 mM ethanol and 100 mM DMSO. Early studies showed that lonidamine selectively inhibits glycolysis in tumor cells and increases cellular acidification by lactate accumulation [72], which led its study as anticancer drug.

### 3.2. Pharmacodynamics

Lonidamine inhibits glycolysis through its inhibitory effect on mitochondrial-bound HK (HK type II). Interestingly, it has been showed that mitochondria-bound hexokinase is more sensitive to lonidamine inhibition than the soluble form of the enzyme (5 *μ*M compared to 75 *μ*M) [73, 74]. The inhibition of HK-II by lonidamine leads to decreased glucose phosphorylation which drops glucose-6-phosphate and reduces, as a consequence, metabolites from glycolysis and pentose phosphate pathways. Lonidamine inhibits lactate production in highly undifferentiated cells from gliomas that have an increase in the activity of this enzyme and leads to cellular acidification by accumulation of lactate via inhibition of lactate efflux [72, 73, 75–77].

Lonidamine causes cell death by apoptosis triggering dissipation of the mitochondrial transmembrane potential, increases reactive oxygen species levels, increases DNA fragmentation, and leads to loss of cell viability. Treatment with inhibitors of apoptosis shows that the* de novo* synthesis of proteins is not needed for the apoptotic effect of lonidamine and that while caspases are downstream effectors for apoptosis, they are dispensable to induce the mitochondrial transmembrane potential reduction [78–80]. The overexpression of the antiapoptotic protein Bcl-2 inhibits lonidamine effects on the mitochondrial membrane, nuclear apoptosis, and cell death. Findings in isolated nuclei indicate that the apoptotic effects of lonidamine are only seen in the presence of mitochondria and that its apoptotic effect is abolished by adding an inhibitor of the permeability transition pore. It is also demonstrated that supernatants of mitochondria treated with lonidamine contain cytochrome c as well as other factors capable of inducing apoptosis. These findings indicate that lonidamine acts through the opening of the mitochondrial permeability transition pore [81]. These observations have been corroborated by other researchers. Belzacq and coworkers found that lonidamine activates the adenine nucleotide translocator (ANT) to form pores and this contributes to the mitochondrial membrane permeabilization [82]. On the other hand, it is known that in mitochondria of cancer cells HKII associates with the voltage-dependent anion channel (VDAC) and this association appears to protect tumor cells from mitochondrial outer membrane permeabilization. It has been shown that the glycolytic inhibitor methyl jasmonate disrupts this interaction [83, 84]. This raises the possibility that lonidamine could also disrupt this interaction; however, this remains to be investigated. In summary, the lonidamine-induced cell death effect is not fully understood but most likely results as a consequence of number of downstream events initiated by the inhibition and/or its interaction with HK-II.

### 3.3. Pharmacokinetics and Metabolism

The pharmacokinetics of lonidamine vary in patients treated with single dose and chronic oral administration but in either case lonidamine is eliminated in the urine by more than 70%. A study by Besner and colleagues found a C infinity max (after drug intake) between 4.5 and 25 *μ*g/mL and a C infinity min (residual plasma concentration before administration) from 0.4 to 7 *μ*g/mL. In this study, concentration levels were related to response, and patients that responded showed a mean value for C infinity min of 2.98 *μ*g/mL whereas the corresponding value for those with no response was 1.5 *μ*g/mL [85].

In a study for chronic administration, 24 breast or lung cancer patients were treated with lonidamine for 27 to 47 days at 150 mg (time 0), 150 mg (*t* = 7 h), and 150 mg or 300 mg (*t* = 14 h). HPLC with fluorescence detection studies revealed an absolute range for the peak plasma levels of 4.6–33.8 and 4.8–33.3 *μ*g/mL for the first and second doses, respectively. The apparent half-life determined in 19 patients ranged between 2.5 and 11.7 hours. Different components were detected; one of them was sensitive to hydrolysis with beta-glucuronidase. There was no relation between lonidamine pharmacokinetics with drug-induced myalgia or testicular pain [86].

Another study by Mansi et al. included 17 patients treated with lonidamine at 600 mg, starting with low doses and increasing during the first week up to 600 mg (150 mg in the morning, 150 mg in the afternoon, and 300 mg at night) during the rest of the month. After one month, blood samples were taken at times 0, 0.25, 0.5, 1, 2, 3, 4, 5, 6, and 7 h after the first and second 150 mg doses and 2 hourly following the 300 mg (third dose). The peak plasma levels of lonidamine after the first 150 mg dose ranged from 7.6 to 33.8 *μ*g/mL (mean 15.5) and after the second from 5.3 to 33.3 *μ*g/mL (mean 15.8). The absolute range of the time at which the peaks were observed was 0.5 to 4.0 h (mean 1.9) for the first and 0.5 to 4.1 h (mean 2.0) for the second dose. The range of plasma half-life was 2.5 to 7.8 h (mean 3.9). These data indicate that lonidamine had been absorbed in all patients. Age correlates with lonidamine pharmacokinetics. Different compounds were found in HPLC analyses of plasma from patients treated with lonidamine, which suggests the drug is metabolized [87].

### 3.4. Clinical Efficacy

Three phase I studies [28–30] showed its tolerability in doses ranging from 180 mg/m^2^ to 520 mg/m^2^. These data led to adopting 450 mg total dose daily for subsequent clinical trials (Table 1). At least 14 phase II studies with lonidamine either as a single agent or in combination with chemotherapy and radiation have been reported in breast, lung, ovarian, and head and neck cancer. The heterogeneity and uncontrolled design of these studies can only suggest the efficacy of lonidamine [36–49] (Table 2). These data led to testing lonidamine in 5 phase III trials in breast [50–54] and 5 trials in lung cancer [55–59]. In breast cancer all the studies report a trend (only one with statistical significance) for higher tumor responses and a trend for better survival parameters in studies combining lonidamine with chemotherapy (Table 3). Similar results were observed in lung cancer. A trend for higher response rates with lonidamine-containing regimens was also observed, though statistically significant differences in response rate, median TTP, and OS were observed only in the trial of cisplatin epirubicin and vindesine with or without lonidamine (Table 4).

### 3.5. Safety and Tolerability

The safety of lonidamine has been demonstrated in hundreds of patients treated. Though there is scarcity of data on phase I studies, a study recommends 135 mg/m^2^ twice daily which can be approximately 660 mg daily in an individual having a 1.7 m^2^ of body surface area. A second study found no limiting toxicity at 520 mg/m^2^ daily, and a third study combining lonidamine with whole body hyperthermia found 360 mg/m^2^ daily as a safe dose. In literature, it has been administered up to 900 mg/day. Because of that, most physicians agreed on 450 mg/day divided into three doses to be recommended dose. Lonidamine has two commonly seen side effects which are mialgias observed up to 60% of patients and testicular pain in up to 27% of patients. Muscular pain starts about 6 hours after administration and typically involves the trunk and lower extremities and tends to decrease with the continuous administration. It has been hypothesized that it originates from accumulation of lactic acid in muscles and there are controversial data on the efficacy of low-dose steroids for its relieve. Testicular pain may occur after prolonged administration that can be due its antispermatogenic effects. Ototoxicity that yet occurs in no more than 10% of patients is characterized by altered perception of speech but is not accompanied by alterations in audiography. It subsides with continued drug administration. Other common effects are gastrointestinal (nausea, vomiting, and epigastralgia) in 24% of patients and asthenia in 16% of patients. Other less common and usually mild effects occurring in <10% of patients are arthralgia hyperesthesia, neurological disturbances, photophobia, skin rash, drowsiness, anorexia, fever, diarrhea, and headache. Of note, lonidamine does not produce myelosuppression even at higher doses (up to 900 mg day) and do not increase the toxicity of classical cytotoxics or radiation [88].

## 4. DON (6-Diazo-5-oxo-L-norleucine)

### 4.1. Chemistry

6-Diazo-5-oxo-L-norleucine also known as DON was initially described as an antitumor antibiotic isolated from an unidentified streptomyces strain. It has a molecular weight of 171.15 and its empirical formula is C_6_H_9_N_3_O_3_. DON is very sensitive to heat and pH, and the optimal pH at room temperature ranges from 4.5 to 6.5. DON is a light yellow powder very soluble in water and aqueous solutions of methanol, acetone, or ethanol [89]. As an analog of glutamine, DON has been used as an inhibitor of glutamine utilizing enzymes such as carbamoyl phosphate synthase (CAD), CTP synthase (CTPS), FGAR amidotransferase, guanosine monophosphate synthetase (GMPS), PRPP amidotransferase, NAD synthase, asparagine synthase, and glutaminase. These enzymes are used in several important metabolic pathways such as the purine, pyrimidine, and amino acid synthesis as well as a coenzyme of the electron transport chain and in the first step of glutaminolysis [89–95].

### 4.2. Pharmacodynamics

The first descriptions of DON as an antitumor agent were published in 1956 by Coffey et al. [96]. Despite its ability to inhibit a number of glutamine utilizing enzymes, its glutaminolytic effect has attracted more attention as it is now recognized as a common feature of most tumor cells. Up to date, three isoforms of human glutaminase have been identified: kidney-type (GLS1), the splice KGA variant (GLS C), and liver-type (GLS2) [97]. A recent study using a crystal structure of the catalytic domain of GLS1 complexed with DON reported that it covalently binds with the active site Ser286 and interacts with residues such as Tyr249, Asn335, Glu381, Asn388, Tyr414, Tyr466, and Val484. The nucleophilic attack of Ser286 side chain on DON releases the diazo group (N2) from the inhibitor and results in the formation of an enzyme-inhibitor complex. This model was confirmed by mutants at the active site region [98].

There are few preclinical studies investigating the antitumor effects of DON. In early studies, DON alone or in combination with antimetabolites was able to downsize mammary murine tumors* in vivo* [99]. DON has also shown remarkable activity in murine tumors growing in mice including murine leukemia L1210 and P388, the colon C26 and C38 and mammary tumor CD8F1, and human mammary MX-1, lung LX-1, colon CX-1, and CX-2 tumors [100], and in an* in vitro* study DON exhibited as much as 10 times more cytotoxicity upon these two murine leukemia as compared to normal embryonic fibroblasts [31]. The glioma cell lines D-54 and MG and the medulloblastoma cell line E-671 are also being tested with DON and shown to be sensitive to the drug [101]. In a study which analyzed the effect of DON upon the cell cycle distribution, it was found that this drug causes a striking S-phase block with concurrent increase in G1 and G2-M populations. Of note the effect was differential between the malignant cell lines tested (Redmond colon tumor, A549 lung, CX-1 and CX-2 colon, and LX-1 lung tumor) as compared to normal human embryonic lung fibroblasts IMR-90 [102]. Studies performed in a neuroblastoma cell line showed that DON targets mitochondria, reporting disruption of mitochondrial internal membrane structures and also the alteration of other organelles such as swelling of endoplasmic reticulum, autophagocytosis of secretory granules, and nuclear condensation or apoptosis [103]. Neuroendocrine cells have been reported to be exquisitively sensitive to DON which induces marked growth inhibition even in cells growing in aggregates which was accompanied by decreases in the secretion of chromogranin A [104]. In neuroblastomas and Ewing's sarcoma cell lines which commonly express c-myc and are addicted to glutamine, DON has potent antitumor activity* in vitro* and* in vivo* [105]. DON was evaluated in the VM-M3 murine model of metastasis where systemic treatment led to profound decrease in tumor proliferation and inhibition of visceral metastases [106]. Interestingly, DON appears to have antiangiogenic activities. In ascites tumor bearing Swiss mice induced by transplantation of Ehrlich ascites cells, DON reduces the secretion of VEGF in the tumor cells treated* in vitro* [107].

### 4.3. Pharmacokinetics and Metabolism

DON is absorbed by the intestinal tract, but due to its acid-labile properties the more suitable administration is intravenously. Apparently, DON does not interact with plasma proteins such as albumin. DON pharmacokinetic parameters are not well established. According to previous studies it seems that DON exhibits dose-dependent pharmacokinetics in adults, as with increased doses of the compound, its clearance decreases and its half-life increases. DON at 300 mg/m^2^ administered as a 10 min i.v. injection had a half-life of 76.2 minutes and clearance of 3.39 mL/min/Kg. The volume of distribution was 449 mL/Kg, which suggests great extravascular distribution [108]. At that dose, DON was not detected in urine samples at 24 hours posttreatment. Other studies showed DON is excreted mainly in urine [32]. The number of patients impedes concluding about the pharmacokinetic parameters in adults. In children treated with DON at different doses from 150 to 520 mg/m^2^, the drug had a half-life between 150 and 177 ± 20 minutes, clearance of 163 to 215 ± 73 mL/min, and volume of distribution between 26 and 44 ± 23 L. Linear correlation of the results in children indicates a positive correlation between clearance and age and volume of distribution and age. DON does not cross the cerebrospinal blood barrier in children. The rapid disappearance from blood involves not only blood cells [33]. Recently, a bioanalytical method to quantify DON in tissue samples has been described involving DON derivatization with 3N HCl in butanol. The derivatized product is lipophilic and stable. Detection of this analyte by mass spectrometry is fast and specific and can be used to quantify DON in plasma and brain tissue with a limit of detection in the low nanomolar level. The results of this preclinical study in mice using a comparable dose used in humans found a half-life of DON of 1.2 hours which is similar to that found in the previous clinical studies using less sensitive methods. Interestingly, the study found brain tissue concentrations of DON plasma/brain ratio of 0.1 which suggest that DON readily crosses the brain barrier [109].

### 4.4. Clinical Efficacy

Data from five phase I studies demonstrate that DON is safe and the recommended doses as single agent vary according to the schedule: 50 mg/m^2^/day × 5 in 21- or 28-day cycles; 300 mg/m^2^ twice weekly in 21-day cycles; 480 mg/m^2^ daily for 3 days in 21-day cycles; 400 mg/m^2^ by 24-hour infusion in a single day in cycles of 21- or 28-day; and 450 mg/m^2^ twice a week every 2 weeks [31–35] (Table 1). As compared to lonidamine, the clinical development of DON did not go into phase III trials. There are only five phase II studies (lung, colorectal, colorectal, sarcoma, and advanced refractory tumors) four done in the 80s and one more published in abstract form in 2008. As a single agent in these four studies responses were uncommon but disease stabilization was reported up to 53% in colorectal cancer. The most recent study evaluated recombinant PEGylated glutaminase combined with DON demonstrating disease stabilization in more than half of colorectal and lung cancer [60–64], (Table 5).

### 4.5. Safety and Tolerability

From the experience with DON for cancer treatment it can be concluded that it is a safe drug and that the dose-limiting toxicity is nausea and vomiting which varied according to the schedule and dose. Divided doses (every four or six hours) caused more nausea and vomiting than the same dose in single daily administration. Despite the fact that DON in addition to its inhibitory action upon GSL1 has a number of targets involved in DNA synthesis, the resulting myelotoxicity is mild with fast recovery, manifested by leucopenia and thrombocytopenia and never life threatening.

Other toxic effects less frequent are diarrhea, oral mucositis, uremia, and gastrointestinal bleeding. No cutaneous, hepatic, renal, or cardiopulmonary toxicities were observed in adults. Neurological effects are also rare. One patient treated with 600 mg/m^2^ of DON for three days presented marked blurring vision for 48 hours after the treatment and returned to normal. Two patients with Hodgkin's disease had mental changes, which consisted in lethargy and confusion in one patient and the other had maniac, paranoid schizophrenia, and this last patient presented schizoid features before treatment.

In children, nausea and vomiting were prevented or controlled with the use of antiemetics. Urinary toxicity was observed in less than one third of the patients as microscopic hematuria. Some patients presented hepatic toxicity manifested by mild or moderate elevation in transaminases [88].

## 5. Rational for the Combination of Lonidamine and DON

Glucose and glutamine are the most abundant circulating nutrients needed to support the growth and proliferation of all cells, particularly not limited to rapidly dividing cancer cells. It was shown that cells in culture undergo apoptosis if depleted of glutamine or glucose [110] and that in hybridoma cultures, at the midexponential phase of growth, the energy contribution from the catabolism of the two substrates is finely balanced: 55% glutamine and 45% glucose [111].

**Figure 1:**
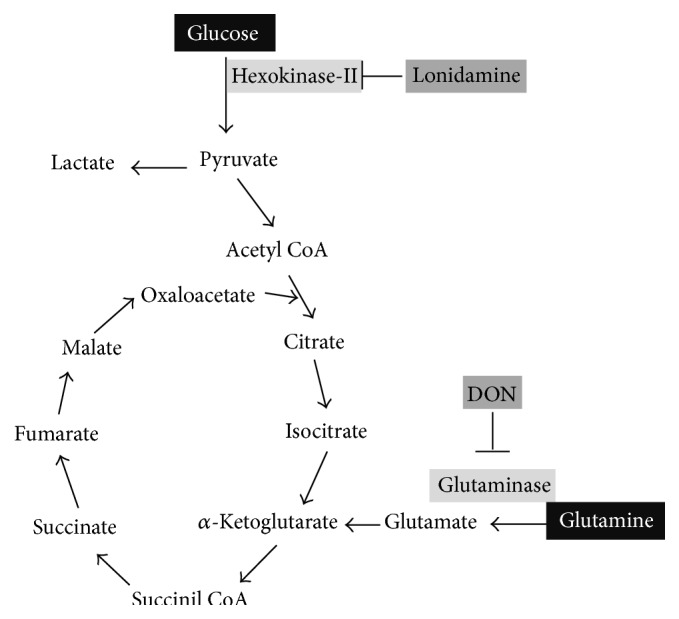
Lonidamine and DON targets and the rational for their combined use. (1) Glucose and glutamine are the key circulating nutrients for proliferating cells. (2) Glycolysis and glutaminolysis are cancer hallmarks. (3) Major oncogenes such as K-ras and c-myc simultaneously regulate glycolysis and glutaminolysis. (4) Cancer cells are primarily glycolytic or glutaminolytic depending upon genetic mutational landscape. (5) Cancer cells are metabolically plastic; hence they can rewire their metabolism upon nutrient availability among other factors. (6) Glycolytic and glutaminolytic inhibitors have antitumor effects on their own. (7) It is expected that upon pharmacological inhibition of glycolysis cells would survive by increasing glutaminolysis and vice versa. (8) Preclinical studies show that the combination of lonidamine and DON results in higher inhibitory effect as compared to each agent by separate. (9) Lonidamine and DON are safe drugs which have been tested in cancer patients.

The importance of glycolysis and glutaminolysis in cancer is well supported by the fact that, in cancer cell lines, oncogenic K-ras exhibited enhanced glycolytic activity, decreased oxidative flux through the tricarboxylic acid cycle, and increased utilization of glutamine for anabolic synthesis [21]. Increased glycolysis and glutaminolysis are also induced by c-myc, another central oncogenic player [112]. The analysis of metabolic flux in multiple tumor cells implicates that tumors are capable of surviving in the nutrient deprived and the hypoxic conditions by collaboratively using glucose and glutamine metabolism, which provide a metabolic platform supporting both bioenergetics and biosynthesis [113]. In this regard, glioma cells treated with 2-deoxyglucose (2-DG), a competitive hexokinase inhibitor, suppress lactate formation and increase glutamine metabolism via activation of GDH (glutamate dehydrogenase) [114]. In the same way, Wu et al. have shown that depletion of PKM2 (pyruvate kinase M2-type), which sustains glycolysis, provokes glutamine metabolism via *β*-catenin and downstream c-Myc and these results can be also observed using the glycolytic inhibitor 2-DG. Treatment with DON of PKM2 knocked-out cells further inhibits malignant cell growth [114]. These works provide evidence that glutaminolysis plays a compensatory role for cell survival upon glucose metabolism impaired. Interestingly, a recent work has also demonstrated that GLS1 (Glutaminase-1) positively regulates glucose uptake in addition to glutaminolysis via transcriptional repression of thioredoxin interacting protein (TXNIP), which is a potent negative regulator of glucose uptake and aerobic glycolysis [115]. This may imply that tumor cells may engage in metabolic compensation* in vivo* to survive in periods of diminished glucose metabolism as suggested by decreases in ^18^FDG-PET signal during cancer therapy which do not necessarily correlate with good outcome [116]. On these facts, glucose and glutamine utilization pathways emerge as natural targets to be simultaneously inhibited given their complementary role in intermediary tumor metabolism.  Figure 1 shows the site of action of lonidamine and DON as well as the rational for its combined use.

On this basis it is surprising that there is only one study performed 22 years ago in which glycolytic and glutaminolytic inhibitors were combined to demonstrate increased antitumor effects. Griffiths et al. showed in the myeloid leukemia cell line THP-1 that DON inhibits the ability of these cells to oxidate but increases lactate production suggesting an increased glycolytic flux. By adding the glycolytic inhibitor 2-DG, lactate production was inhibited which correlated with increased growth inhibition. The increased effect of the combination was also demonstrated in fresh leukemia blast from a patient [117]. We recently demonstrated that the combination of lonidamine and DON plus a fatty acid synthase inhibitor aimed to block three key pathways, glycolysis, glutaminolysis, and* de novo* synthesis of fatty acids, has strong antitumor effects in 13 cancer cell lines as compared to nontransformed cells. When the combination was tested for their pharmacological interaction in the colon cancer cell line SW480, we found a synergistic interaction between them [118]. Interestingly, by assessing the interaction between any pair of these agents, the only synergistic interaction was found with lonidamine and DON at 100 and 25 *μ*M, respectively (unpublished results).

## 6. Conclusions

The study of cancer metabolism has renewed interest since the discovery that major gene and pathway alterations commonly found in cancer affect tumor metabolism. Glucose and glutamine are the main carbon and energy sources for cells, especially for cancer cells that have a high proliferation rate and need building blocks for the new cells and energy. As such, common features to cancer cells are higher rates of glycolysis and glutaminolysis. A number of preclinical studies demonstrate that inhibiting these altered pathways leads to strong antitumor effects; as a consequence there are efforts to develop drugs to target them. Lonidamine and DON are two known drugs that were clinically evaluated as anticancer agents when the therapeutic potential of metabolic inhibitors against glycolysis and glutaminolysis was incipient. From this review it seems clear that these drugs deserve continuing evaluation as they are safe and potentially effective.

The clinical experience with lonidamine is large, demonstrating that it can be safely combined with chemotherapy and radiation because it has nonoverlapping toxicity.

The development of DON was also stopped mainly because of its dose-limiting nausea and vomiting toxicity which nowadays should not be a problem with the availability of potent and effective antiemetics. We cannot conclude with its potential efficacy because the clinical experience was much less as compared with lonidamine. Unfortunately, the clinical development of both drugs was abandoned, most likely from factors related to study designs and underpowered sample sizes in a time where the bar for cancer drugs approval was high [119].

So far, there are no clinical studies combining metabolism-targeting agents to simultaneously block the two most known pathways, glycolysis and glutaminolysis. An early* in vitro* study showed that combination of DON with 2-deoxy-D-glucose led to remarkable inhibition of both glutamine oxidation and glycolysis which was accompanied by increased cytotoxicity against the human TPH-1 myeloid cell line and freshly cultured myeloid blast cultures from a patient. This should not be underestimated; after all, the metabolic phenotype of cancer cells is highly plastic having the ability to change metabolic fluxes according to the availability of nutrients. Thus, it is expected that glycolytic tumors may opt for glutaminolysis to resist glycolytic inhibitors and vice versa; therefore the combination of DON and lonidamine (or any other pair of glycolysis and glutaminolysis inhibitors) is a promising clinical research avenue to explore.

## Figures and Tables

**Table 1:** Phase I clinical studies with lonidamine and DON.

Drug	Number of studies	Number of patients per study	Tumor type	Dose escalation	Recommended dose	References
Lonidamine	3 studies	15, 31, and 24 (70)	Several, advanced	350–400 mg/m^2^ 180–520 mg/m^2^ 60–360 mg/m^2^	450 mg daily, orally	[28–30]

DON	5 studies	26, 26, 25, 21, and 17 (115)	Several, advanced		50 mg/m^2^/day ×5 in 21- or 28-day cycles 300 mg/m^2^ twice weekly in 21-day cycles 480 mg/m^2^ daily for 3 days in 21-day cycles 400 mg/m^2^ by 24-hour infusion in a single day in 21- or 28-day cycles 450 mg/m^2^ twice a week every 2 weeks	[31–35]

**Table 2:** Summary of phase II clinical studies with lonidamine.

Number of studies	# pts	Tumor type	Treatment	Overall response rate (ORR)	Observations	References
4	51	Breast	LND + epi:	21%	Metastatic disease	[36–39]
	24 29 22		LND + epi; LND + epi + cis LND + epi + cis	57% 73% 81.8%	Mean response duration 12.4, 7.0, and 9.8 months in the first three trials, respectively	

3	25 54 31	Lung	LND alone; LND + cis + gem + vnr; LND + cis + epi	24%, 37% 41.4%	Advanced or metastatic disease Mean response duration 13.7, 4.5, and 8.5 months	[40–42]

4	35 27 9 26	Ovarian	LND + cis + pac LND + cis LND + cis LND + epi	80% 37% 44% 33.3%	LND + cis + pac, untreated pts. PFS 28.5 months The other 3 studies in pretreated patients	[43–46]

3	40	Head and neck	LND + RT	CR 65%	Untreated	[47–49]
	89		LND + mtx versus mtx + pla	ORR 26.3 versus 18.2%	Advanced/recurrent	
	96		LND + RT versus RT + pla	CR 66 versus 65%	Untreated 5-year DFS 40 versus 19%, *p* < 0.03	

LND: lonidamine; epi: epirubicin; cis: cisplatin; gem: gemcitabine; vnr; vinorelbine; pac: paclitaxel; RT: radiation; mtx: methotrexate; pla: placebo; CR: complete response; PFS: progression-free survival; DFS: disease-free survival.

**Table 3:** Phase III clinical studies with lonidamine in metastatic breast cancer.

Patients accrued	Treatment	Response %	Observations	References
265	LND versus LND + FAC	ORR (%) 42.3 versus 66.3	Median PFS 6 versus 9 months *p* < 0.0001 Multicentric study	[50]

181	dox to all, then randomized to dox + LND versus dox	ORR (%) 50 versus 38	Response in liver metastases 68 versus 33% *p* = 0.03	[51]

326	FEC/EM versus FEC/EM + LND	CR 10.8% versus 20.4%	No differences in PFS or OS	[52]

207	LND + epi versus epi	ORR (%) 60 versus 39 *p* < 0.01	Higher response in liver metastases with LND + epi. No differences in PFS or OS	[53]

371	epi versus epi + cis versus epi + LND versus epi + cis + LND	ORR (%) 54 no LND arms versus 62.9 LND arms *p* = 0.08	Median OS 29.8 versus 32.2 months TTP 9.9 versus 10.8 months, trend favoring LND arms, *p* = NS	[54]

FAC: 5-fluorouracil-doxorubicin-cyclophosphamide; dox: doxorubicin; FEC: 5-fluorouracil-epirubicin-cyclophosphamide; EM: epirubicin-mitomycin; cis: cisplatin; ORR: overall response rate; PFS: progression-free survival; OS: overall survival; TTP: time to progression.

**Table 4:** Phase III clinical studies with lonidamine in lung cancer.

Patients accrued	Treatment	Main findings	Observations	References
184	LND versus mit + vds versus LND + mit + vds	ORR (%) 3.4% versus 22.4% versus 25.9% *p* < 0.01	1-year OS rate mit + vds 20% mit + vds + LND 32%	[55]

158	cis + epi + vds versus cis + epi + vds + LDN	ORR (%) 24 versus 43 *p* = 0.02	*Median TTP* *5 *v*ersus 8 m. p* = 0.0007 *Median OS* *7.6 versus 11 m. p* = 0.0013	[56]

151	MACC versus MACC + LND	ORR (%) 7 versus 13	Median PFS 17 versus 20 weeks *p* = NS Median OS 27 versus 30 weeks *p* = NS	[57]

126	LND versus vds versus LND + vds versus BSC	ORR (%) LND 3.3 LDN + vds 6	Elderly patients Median OS all pts 24.2 weeks No differences among regimens	[58]

310	LND + RT versus pla + RT	More local control in LND + RT arm (*p* = NS)	Median PFS 7.6 versus 6.5 months, *p* = 0.75 Median OS 13 versus 10.8 months *p* = 0.41	[59]

mit: mitomycin-C; vds: vindesine; MACC; methotrexate-doxorubicin-cyclophosphamide-CCNU; BSC: best supportive care; ORR: overall response rate; PFS: progression-free survival; OS: overall survival; TTP: time to progression.

**Table 5:** Phase II studies with DON.

Number of patients	Tumor type	DON schedule	Observations	References
22	Advanced lung cancer	160 mg/m^2^ days 1–3, every 21 days	4 (18.1%) had transient disease stabilization (3–12 weeks)	[60]

30	Advanced colorectal	160 mg/m^2^ days 1–3, every 21 days	16 (53%) had disease stabilization (3–26 weeks)	[61]

23 (14 evaluable)	Advanced colorectal	300 mg/m^2^ days 1–5 every 2 weeks (deescalated to 200 mg)	PR 1 (7%) SD 2 (14%), 2 and 5.5 months	[62]

36	Pretreated advanced sarcoma	50 mg/m^2^ days 1–5, every 28 days	No responses median OS 4.8 months	[63]

55	Advanced, refractory malignancies	140 mg/m^2^ twice weekly + PEG-PGA	Treatment delivered 1–379 days 1 PR and 1 SD (>12 months) in 17 colorectal pts. 5 of 6 with lung cancer SD in the first 3 months of inclusion	[64]

PEG-PGA: PEGylated recombinant human glutaminase; PR: partial response; CR: complete response; SD: stable disease; OS: overall survival.
